# Investigating the effect of modifying the EEG cap lead configuration on the gradient artifact in simultaneous EEG-fMRI

**DOI:** 10.3389/fnins.2014.00226

**Published:** 2014-07-29

**Authors:** Karen J. Mullinger, Muhammad E. H. Chowdhury, Richard Bowtell

**Affiliations:** ^1^Sir Peter Mansfield Magnetic Resonance Centre, School of Physics and Astronomy, University of NottinghamNottingham, UK; ^2^Birmingham University Imaging Centre, School of Psychology, University of BirminghamBirmingham, UK

**Keywords:** simultaneous EEG-fMRI, gradient artifact, optimizing wire configuration, artifact reduction, head geometry

## Abstract

EEG data recorded during simultaneous fMRI are contaminated by large voltages generated by time-varying magnetic field gradients. Correction of the resulting gradient artifact (GA) generally involves low-pass filtering to attenuate the high-frequency voltage fluctuations of the GA, followed by subtraction of a GA template produced by averaging over repeats of the artifact waveforms. This average artifact subtraction (AAS) process relies on the EEG amplifier having a large enough dynamic range to characterize the artifact voltages and on invariance of the artifact waveform over repeated image acquisitions. Saturation of the amplifiers and changes in subject position can leave unwanted residual GA after AAS. Previous modeling work suggested that modifying the lead layout and the exit position of the cable bundle on the EEG cap could reduce the GA amplitude. Here, we used simulations and experiments to evaluate the effect of modifying the lead paths on the magnitude of the GA and on the residual artifact after AAS. The modeling work showed that for wire paths following great circles, the smallest overall GA occurs when the leads converge at electrode Cz. The performance of this new cap design was compared with a standard cap in experiments on a spherical agar phantom and human subjects. Using gradient pulses applied separately along the three Cartesian axes, we found that the GA due to the foot-head gradient was most significantly reduced relative to a standard cap for the phantom, whereas the anterior-posterior GA was most attenuated for human subjects. In addition, there was an overall 37% reduction in the RMS GA amplitude produced by a standard EPI sequence when comparing the two caps on the phantom. In contrast, the subjects showed an 11% increase in the average RMS of the GA. This work shows that the optimal design reduces the GA on a spherical phantom however; these gains are not translated to human subjects, probably due to the differences in geometry.

## Introduction

Electroencephalography (EEG) data recorded simultaneously with functional Magnetic Resonance Imaging (fMRI) acquisition is becoming a widely used technique for studying brain function (e.g., Mayhew et al., 2013; Mullinger et al., 2013c; Walz et al., 2013; Warbrick et al., 2014). The complementary temporal and spatial information which can be obtained from the two techniques enables more information to be acquired about the brain than either technique can provide alone. Combined EEG-fMRI therefore opens up opportunities for developing a better understanding of brain function and of the origin of the haemodynamic signals measured in fMRI. The promise of this technique, combined with the commercial availability of MR-compatible EEG systems, means that simultaneous EEG-fMRI is now being used by neuroscientists in answering numerous research questions (e.g., Plichta et al., 2013; Walz et al., 2013; White et al., 2013; Hauser et al., 2014).

EEG data acquired during simultaneous fMRI is, however, confounded by a number of artifacts which swamp the neuronal signals of interest. The largest of these artifacts arises from voltages generated by the time-varying magnetic field gradients (Allen et al., 2000). The resulting gradient artifact (GA) can be more than three orders of magnitude larger than the signals of interest from the brain (Mullinger et al., 2011). The other dominant artifacts are the pulse artifact, linked to the subject's cardiac cycle (Debener et al., 2008; Mullinger et al., 2013b), and motion artifacts, caused by movement of the EEG equipment in the MR scanner due to subject motion or vibration (Eichele et al., 2010). These artifacts severely corrupt the EEG data and without post-processing methods render it impossible to investigate the neuronal EEG signals of interest. Correction of EEG artifacts is therefore essential when combining EEG and fMRI data which have been acquired simultaneously.

The inherent periodicity of the gradient artifact (GA) related to the known, or measurable, timings of the artifact occurrences makes this artifact easy to correct in principle. Most methods for correcting this artifact rely on forming a GA template by averaging over many repeats of the GA waveforms and then subtracting this template from each occurrence of the GA in the EEG data (Allen et al., 2000). To ensure the successful implementation of this average artifact subtraction (AAS) correction method the artifact waveform must be precisely sampled, have a smaller magnitude than the dynamic range of the EEG amplifier and be stable over image acquisitions. The first condition can be satisfied through synchronization of the EEG and MR scanner clocks (Mandelkow et al., 2006; Mullinger et al., 2008b), while the current practice for limiting the magnitude of the GA is to use a low-pass hardware filter to attenuate the large, high-frequency voltage fluctuations produced by the gradient waveforms (Mullinger et al., 2013a). To achieve invariance of the artifact waveform, the subject must be stationary over the entire data acquisition, as any subject movement alters the morphology of the induced GA and therefore compromises the efficacy of AAS.

As described above, in the absence of filtering, the GA induced on an EEG lead can easily be more than 100 mV in magnitude which would require an extremely large dynamic range and a large number of bits for digitization (Mullinger et al., 2011). Fortunately a low-pass hardware filter can be used to attenuate the GA because the dominant contributions to the power spectrum of the artifacts occur at frequencies that are much higher than those of the neuronal signals. This filter is usually set to have a 250 Hz cut off which is satisfactory for the majority of studies as most neuronal activity occurs at frequencies lower than this value. This filter ensures the artifacts are typically reduced in magnitude by at least a factor of 10 and therefore allows a lower dynamic range to be employed whilst avoiding the saturation of the amplifier. However, this cut-off limits the accuracy with which the artifact can be sampled and also prevents the study of ultra-high frequency neuronal activity (Freyer et al., 2009). In addition, even with such a hardware filter it is still possible to saturate EEG channels under some circumstances, thus preventing artifact correction and with the development of higher performance gradient systems this problem is likely to be exacerbated in the future.

As already alluded to there are additional problems in GA correction if the subject moves during data acquisition. Variations in subject position result in changes in the location of the EEG leads and electrodes relative to the magnetic field gradients produced by the MRI scanner. As a result the amplitude of the induced GA varies over volume acquisitions when movements occur. Consequently any movement significantly reduces the efficacy of AAS in removing the GA. A number of post-processing methods have been developed to improve the performance of AAS when subject movement has occurred during data acquisition. The simplest of these involves using a sliding-average whereby only a sub-set of the volumes (typically around 50) closest in time of acquisition to the volume to be corrected are used in forming the average (Allen et al., 2000; Becker et al., 2005). Moosmann et al. proposed an extension of this simple approach that entails using the fMRI motion parameters to guide the formation of the artifact templates (Moosmann et al., 2009), while Freyer et al. have presented a method by which the morphology of each occurrence of the artifact is compared with all others in the data set and those which are most similar are then used to create a weighted average template to correct that specific artifact occurrence (Freyer et al., 2009). For any of these AAS methods where a sub-set of volumes are used for GA correction, correctly choosing the number of volumes to average can pose difficulties: too few volumes can result in the template containing, and therefore removing, neuronal activity, while inclusion of too many volumes can mean that residual GAs remain (Mullinger et al., 2008a). As a result, large residual GA at the higher frequency range of the 250 Hz band often remain after AAS. These are commonly removed by applying additional low-pass filtering after artifact correction with a cut-off frequency around 80 Hz (e.g., Benar et al., 2007; Mayhew et al., 2010; Plichta et al., 2013; Hauser et al., 2014). The presence of residual GAs combined with the low amplitude of neuronal activity at high frequencies makes studying gamma activity (30–100 Hz) inside the MR scanner environment particularly challenging with current methods (Ryali et al., 2009). In addition, study of ultra-high frequency neuronal activity during fMRI currently requires the EEG and fMRI data acquisitions to be interleaved by use of a stepping stone approach (Anami et al., 2003; Freyer et al., 2009). This involves the use of customized fMRI sequences which are generally not available to all investigators.

It is clear that a reduction of the magnitude of the raw GA during data acquisition would be advantageous. A reduction in the GA amplitude would allow the filter bandwidth to be increased in order to facilitate acquisition of higher frequency neuronal signals without saturation of the EEG amplifiers (Freyer et al., 2009). Alternatively the reduced artifact magnitude at a standard 250 Hz cut-off frequency would reduce the demands on the amplifier's dynamic range, thus allowing the voltage resolution to be increased at a fixed number of digitization bits.

By providing an improved understanding of the origin of the GA, previous simulation work suggested a number of ways by which the amplitude of the GA could be reduced during data acquisition (Yan et al., 2009). One suggestion was to change the subjects' axial head position relative to the scanner's isocentre. This approach was subsequently shown to reduce the root-mean-square (RMS) amplitude of the GA significantly (Mullinger et al., 2011). In particular, it was shown that there was an overall 40% reduction in the raw GA, and a 36% reduction of the residual GA after artifact correction for recordings made at the optimal position (electrodes Fp1 and Fp2 at isocentre) compared with a standard position (nasion at isocentre). Simulations also indicated that the GA might be further reduced by changing the EEG cap lead layout and the position of the cable bundle on the cap (Yan et al., 2009). This stemmed from the realization that the GA results from a superposition of the voltages induced in the EEG leads and those produced at the surface of the head, which means that there is potentially an optimal lead configuration in which the lead voltages maximally cancel those induced at the surface of the head. In the study reported here, we explored this concept through simulations and experimental investigation on both phantoms and subjects. We first used simulations to evaluate the effect of modifying the lead paths on the magnitude of the GA and then fabricated a new EEG cap based on the optimal lead configuration identified from the simulations. We then compared the GA produced using the new cap design and a standard cap design and also evaluated the residual artifact after AAS for both designs.

## Methods

### Simulation

All simulations and data analysis were carried out in Matlab using programmes written in-house. The numerical calculations required for the simulations were performed using previously described methods (Yan et al., 2009). Briefly, these calculations involved calculating the contribution of the temporal derivative of the vector potential (−dA/dt) to the induced artifact by line integration along each lead path, with the value from the reference lead subtracted from the values calculated from other leads. Using the assumption that the head can be represented as a spherical volume conductor with the electrodes on the surface, the scalar potential at each electrode was then calculated and the value at the reference electrode subtracted. The vector and scalar potential contributions were then added to give an estimate of the GA measured on each lead. To allow comparison of the performance of the modified cap designs with that of a standard 32 channel EEG cap, the real lead paths on a standard EEG cap when placed on a human head were digitized using a Polhemus Isotrak system, as previously described (Yan et al., 2009), Figure 1B. This cap contained 30 electrodes following the extended international 10–20 system with the reference electrode placed at FCz. In addition, the cap carried electrooculography (EOG) and electrocardiography (ECG) leads whose paths were not digitized or modeled. The electrode positions on the standard cap were projected onto the spherical surface, which best fitted the human head on which the electrodes were digitized. This projection ensured the assumptions made to calculate the scalar potentials in the numerical simulations were satisfied and this also allowed the modified electrode positions to be used as the start point of each of the lead paths when calculating the induced voltages for the lead configurations of the novel cap arrangement.

**Figure 1:**
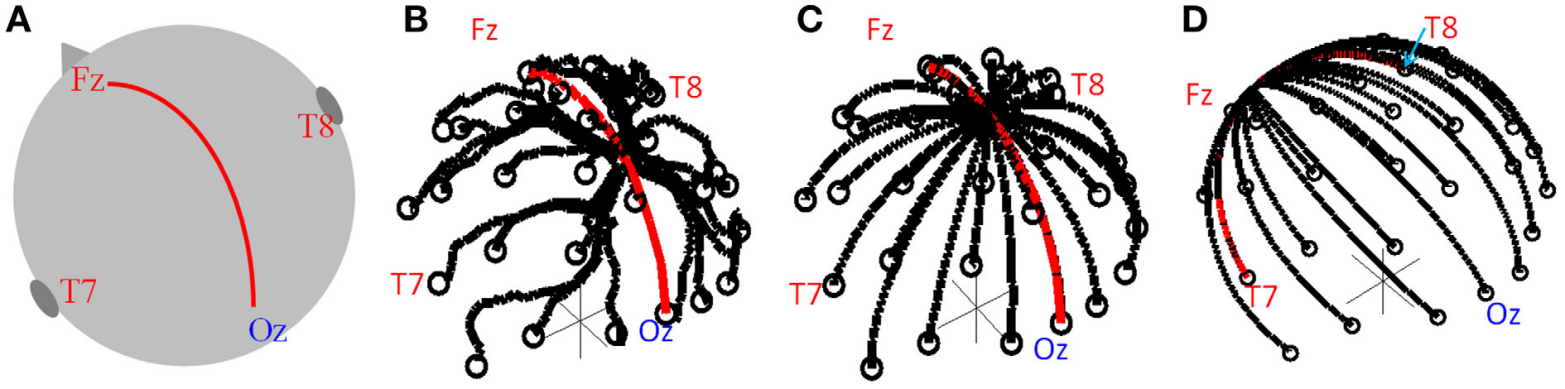
**(A)** Schematic of head showing the midline path along which the convergence point was moved. Lead paths used in simulation for the standard cap **(B)** and modified caps **(C,D)**. The convergence point was moved along the midline (**C**, red line) and along the great circle running right-left (**D**, red line).

To find the theoretical optimal lead configuration the head was again modeled as a sphere and the lead paths formed great circles so as to minimize the length of the leads on the cap (which would be expected to limit the magnitude of the GA). Each great circle started from an electrode position, with all great circles converging to a point from which the cable bundle leaves the cap, as shown in Figure 1C. The electrode at FCz was chosen to be the reference electrode, reflecting this aspect of the standard cap set-up. As in the previous work (Yan et al., 2009) we assumed no contribution to the GA from the wires in the cable bundle since the induced artifacts in the leads serving each electrode and the reference lead should be equal and opposite in the cable bundle; therefore these contributions should cancel. The GA in a series of lead configurations were evaluated as the position of the convergence point of the leads (i.e., the cable-bundle exit position) was moved along the midline following a great circle between electrodes Fz and Oz (Figures 1A,C, red line). The convergence point was also moved in a right-left direction along the great circle between electrodes T7 and T8 (Figure 1D, red line). 100 possible positions of the lead convergence point along each of these two great circles were tested.

The GA induced by gradients applied in the Anterior-Posterior (AP), Right-Left (RL) and Foot-Head (FH) directions were calculated for the standard and modified lead configurations. The range and root-mean-square (RMS) amplitude of the induced GA across electrodes were calculated for each configuration. The simulations were performed with the center of the sphere at the scanner's iso-center along the x- and y-axis. Considering the axial position, electrodes Fp1 and Fp2 were positioned at *z* = 0, corresponding to the 4 cm shift of the head in the foot-head direction that was previously shown to minimize the artifact for a standard cap arrangement (Mullinger et al., 2011). The results of the GA simulations for the different convergence points were compared and the optimal location for the cable bundle to leave the cap found. The results of this optimally configured modified cap were compared with GA simulations using the real lead paths from a standard EEG cap, as previously described (Yan et al., 2009).

### Experimental

**Figure 2:**
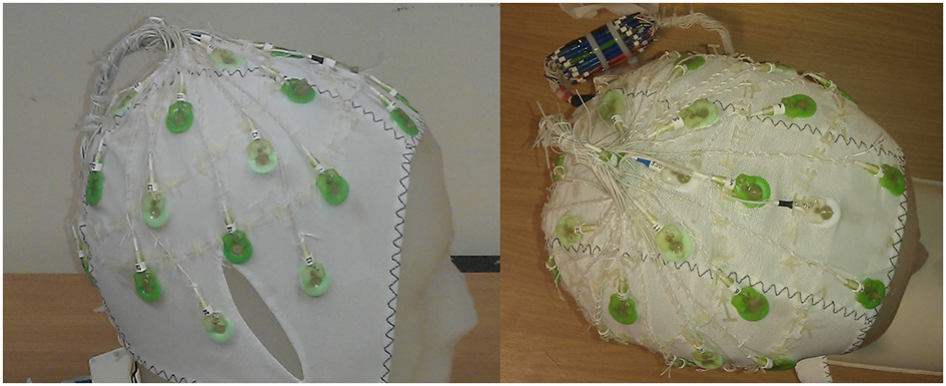
**Photos showing the optimal cap design with each wire sewn onto the cap**.

EEG data were recorded in a 3 T Philips Achieva MR scanner (Philips Medical Systems, Best, Netherlands) using two different 32-electrode EEG caps, a BrainAmp MR-plus EEG amplifier and Brain Vision Recorder software (Brain Products, Munich, Germany). The EEG amplifier was set to a sampling rate of 5 kHz, with the EEG clock synchronized to the MR scanner clock to ensure consistent sampling of the GA waveforms (Mandelkow et al., 2006; Mullinger et al., 2008b). The electrode positions for both the standard and modified EEG caps followed the extended international 10–20 electrode configuration, which was also used in the simulations. The *standard EEG cap* employed a standard lead configuration, as provided by the manufacturer (EASYCAP, Herrsching, Germany), with the cable bundle leaving the cap mid-way between electrodes Cz and Pz, as shown in Figure 1B. The *modified EEG cap* used the optimal lead paths and cable bundle position identified from the simulations, with the cable bundle leaving the EEG cap at Cz, as shown in Figure 1C. To ensure the optimal lead paths in the experimental set-up followed the simulated ones as closely as possible, each lead was sewn to the cap along its entire length, with leads in the cable bundle twisted tightly together (Figure 2).

Artifact voltages were first measured using a 19-cm-diameter, saline-loaded spherical agar phantom (Yan et al., 2009). Subsequently, GA voltages were recorded on five subjects, with approval of the local ethics committee and informed consent. The order in which the data were acquired for the two caps was randomized over subjects. Data from both caps were acquired in the same experimental session for each subject, with subjects washing and drying their hair between cap applications in order to remove all of the conductive gel used in the first set of recordings. Since subject positioning is known to effect GA amplitude (Mullinger et al., 2011), 9 and 7 repeats of the measurements were made for Studies 1 and 2, respectively, on the phantom, and 3 repeats of each measurement were made on the subjects for each cap. The phantom/subject was removed from the scanner and head coil and then returned to the head coil and scanner between successive measurements. Positioning was kept as similar as possible across repeats and subjects, with electrodes Fp1 and Fp2 placed axially at iso-center each time (Mullinger et al., 2011) and the phantom/subjects were also centered in the right-left axis. A twisted cable bundle running down the entire length of the scanner bore, rather than a ribbon cable, was used in order to minimize the induced GA from the cabling connecting the cap to the EEG amplifiers (Chowdhury et al., 2012). The cable bundle was mounted on a cantilevered beam running along the axis of the bore so as to minimize any GA variability due to vibration of the cabling (Mullinger et al., 2013a).

All data analysis for the following experiments was carried out in Brain Vision Analyzer2 (version 2.0.2.5859) (Brain Products, Munich, Germany) and Matlab.

#### Study 1: orthogonal gradients

To identify the effect of lead paths on the magnitude of the GA produced by the three orthogonal gradients, EEG recordings were made during execution of a modified EPI sequence in which gradient pulses, ramping up and down with a slew rate of 2 Tm^−1^s^−1^, were sequentially applied in the RL, AP and FH directions prior to each slice acquisition (Mullinger et al., 2011). The sequence was repeated 30 times and the filters, which limit the frequency range of the recorded EEG data, were set to 0.016–1000 Hz with a roll-off of 30 dB/octave at high frequency to ensure accurate characterization of the induced GA. To measure the artifact voltage on each channel we employed methods previously described (Mullinger et al., 2011). In brief, the GA induced by each of the pulses was averaged over the central 5 ms of each 10 ms ramp period for the 30 repeats. The difference between the voltages induced during the ramp-up and down periods for each of the pulses was used as a measure of the induced GA, which was independent of baseline and high frequency fluctuations. The range and RMS amplitude of the artifacts across electrodes were then calculated for each cap and gradient direction and averaged over repeats and subjects.

#### Study 2: EPI

In the second study, EEG data were recorded during the execution of a standard axial, multi-slice EPI sequence (*TR* = 2 s, *TE* = 40 ms, 84 × 84 matrix, 3 × 3 mm^2^ in-plane resolution, 4 mm slice thickness, flip angle = 85°, fold-over direction = AP, SENSE factor = 2,—i.e., a two-fold reduction in the number of lines of k-space acquired). Twenty slices were acquired with equidistant temporal spacing in each TR-period, such that the frequency of slice acquisition was 10 Hz. This standard sequence allowed the evaluation of the effect of the lead paths and cable configuration on the GA induced by a sequence that is conventionally used for simultaneous EEG-fMRI. 50 volumes of data were acquired on the phantom, whilst 185 volumes were acquired on the human subjects. To mimic the movements that may occur during longer EEG-fMRI runs and thus allow evaluation of the effect of small movements on the GA, the phantom was manually rotated and the subject cued to move their feet for 5 s every 30 s with a total of 10 movement periods in each data acquisition, following a protocol used in a previous study (Mullinger et al., 2011). The EEG data were recorded with a frequency range of 0.016–250 Hz in this study. This is typical of the bandwidth that is used in EEG-fMRI studies in order to avoid saturating the EEG amplifiers.

Data were exported to Matlab for analysis of the GA both before and after artifact correction had been carried out using AAS in Brain Vision Analyzer2. The AAS-template spanned one TR period and was formed from an average of the GA over the entire acquisition period. This long averaging period ensured maximum sensitivity to changes in the GA due to the movements. No down-sampling or filtering of data were employed so as to allow the GA signals over the entire frequency range to be evaluated.

To assess the effect of the EEG cap lead configuration on the induced GA before artifact correction, the artifact waveforms for each slice acquisition (100 ms in duration) for each lead were baseline corrected (relative to the average over the entire 100 ms period) and averaged over all slices (excluding periods when movements were present) within an acquisition period. The RMS over the slice acquisition period was then calculated for the average artifact on each lead. An average over leads and repeats was then taken for the phantom and subject recordings. For the subject data, the average and standard deviation over subjects was also calculated. A Wilcoxon signed-rank test was applied to the subject data to assess if there were significant differences between the GA induced by the EPI sequence for the two different lead configurations.

To evaluate the effect of the lead configuration on the residual GA after AAS, we focused on signals occurring at harmonics of the 10 Hz slice repetition frequency in the range 0–250 Hz. Data were filtered in Brain Vision Analyzer2 so as to pick out signal contributions falling within a 0.1 Hz frequency range around each harmonic. The RMS amplitude of these signals over the entire acquisition period was calculated and averaged over channels and repeats for both the phantom and subject data using Matlab. The average and standard deviation over the subjects was also calculated. A Wilcoxon signed-rank test was applied to the subject data to test for significant differences in the residual GA after artifact correction between the two different lead configurations.

To test whether head/phantom movements were similar across repeats, data were realigned using SPM8 (http://www.fil.ion.ucl.ac.uk/spm/). The RMS of the mean corrected realignment parameters (x, y, and z translation and pitch, yaw and roll) were calculated for each data set and the average and standard deviation of this measure over repeats found.

## Results

### Simulation

**Figure 3:**
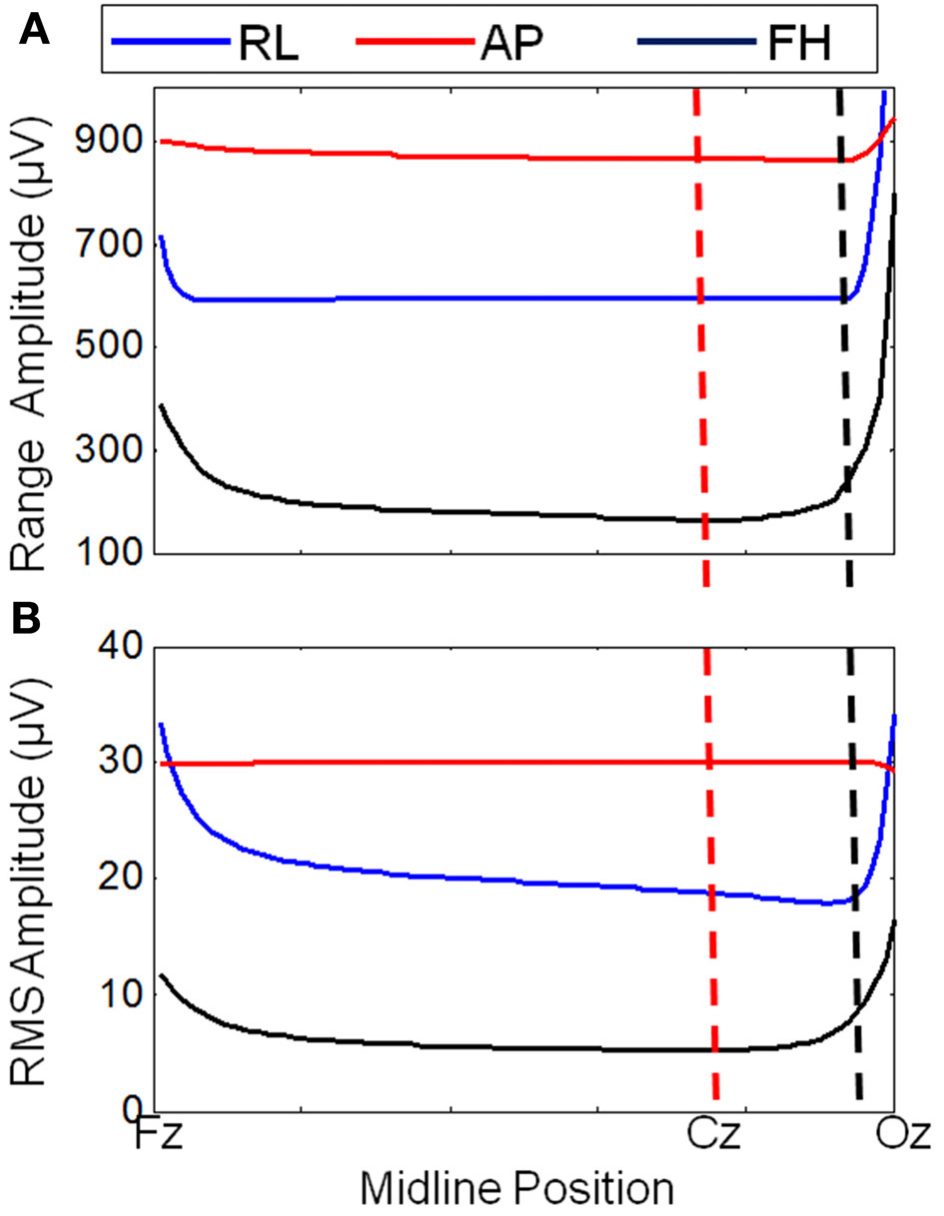
**Variation of the range (A) and RMS (B) of the average GA (over electrodes) with cable bundle position (along the midline) for simulated RL, AP, and FH gradients**. The dashed lines indicate the cable bundle position on standard (black) and optimally modified (red) caps.

**Figure 4:**
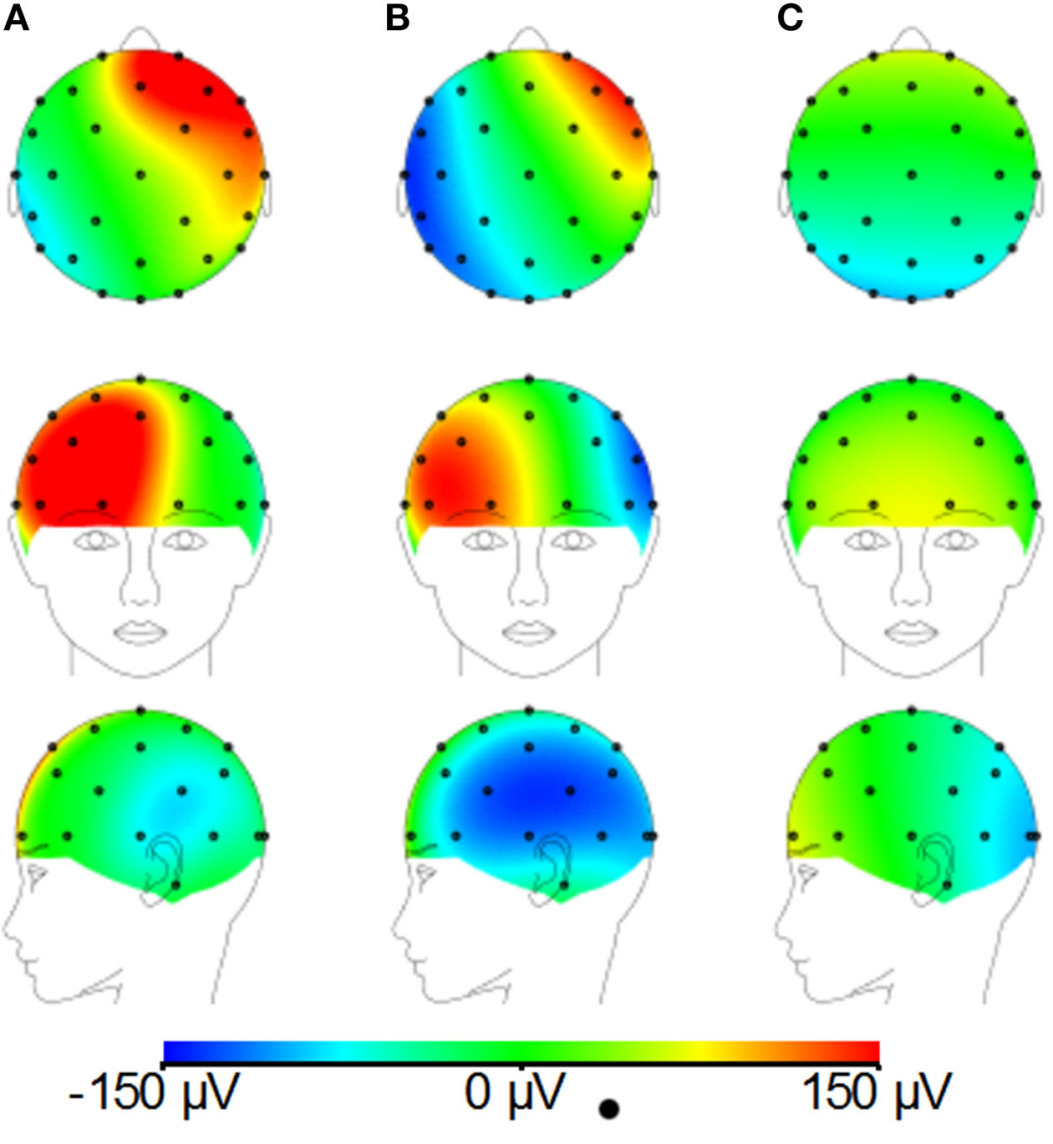
**Simulated artifact maps for a FH gradient at 2 Tm^−1^s^−1^ for real lead paths (A), great circle lead paths converging in the conventional location (between electrodes Cz and Pz) (B) and great circle lead paths converging at the optimal location (electrode Cz) (C)**.

Figure 3 shows that changing the lead paths by moving the cable bundle position along the midline produces a decrease in the range of the GA for the FH gradient, but little change for the RL or AP gradients compared with the conventional position of the cable bundle (Figure 3A, black dashed line). The RMS measure showed a similar behavior, although using this measure a measureable change in the GA induced by the RL gradient was also observed (Figure 3B). From these simulations, a sensible compromise position was found to be with the cable bundle at the position of electrode Cz, as depicted in Figure 1C and denoted by the red dashed line in Figure 3. Figure 4 shows the spatial patterns of the GA induced by the FH gradient for three different lead arrangements. Figure 4 shows the numerical data produced when real lead paths are employed (Figure 4A), compared with the patterns generated when the leads follow great circles converging on a cable bundle located at the conventional position (Figure 4B), and at the optimal position, Cz (Figure 4C). This figure shows that for the FH gradient, varying the lead paths and the convergence point of the cable bundle not only reduces the amplitude of the induced artifact, but also changes the spatial pattern to give an anterior-posterior (Figure 4C) rather than right-left (Figures 4A,B) GA pattern of variation. Consequently, the simulations suggest that the greatest reduction in the GA induced by a FH gradient for the modified cable configuration will occur over the temporal regions, but that this will be accompanied by an increase in the artifact induced over the anterior and posterior regions of the head.

**Figure 5:**
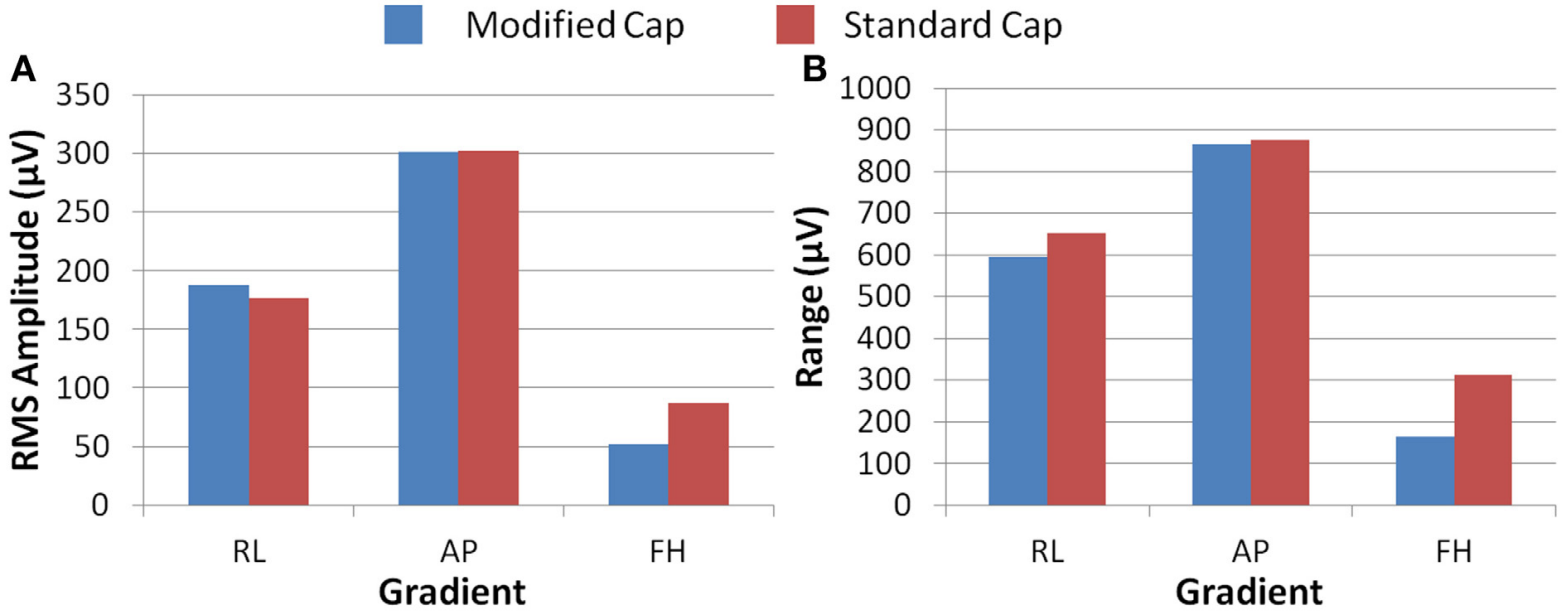
**The RMS (A) and range (B) of the simulated induced GA over all electrodes for each orthogonal gradient (RL, AP, and FH); for the modified (blue) and standard (red) lead paths**.

Figure 5 shows the RMS and range over all electrodes of the GA induced by the three different gradients. These data were derived from the numerical simulations using the real and modified lead paths (Figures 1B,C, respectively). When compared with real lead paths, the modified cap design shows a decrease in the range of the simulated GA of 48, 1, and 9% for the FH, AP and RL gradients respectively. The RMS of the simulated GA showed a 40% decrease for the FH gradient, no change for the AP gradient and a 6% increase for the RL gradient when the results for the modified cap were compared with the real lead paths. Moving the cable bundle away from the midline in the RL direction, as shown in Figure 1D, did not reduce the GA measures for any of the applied gradients.

### Experimental

#### Study 1: orthogonal gradients

**Figure 6:**
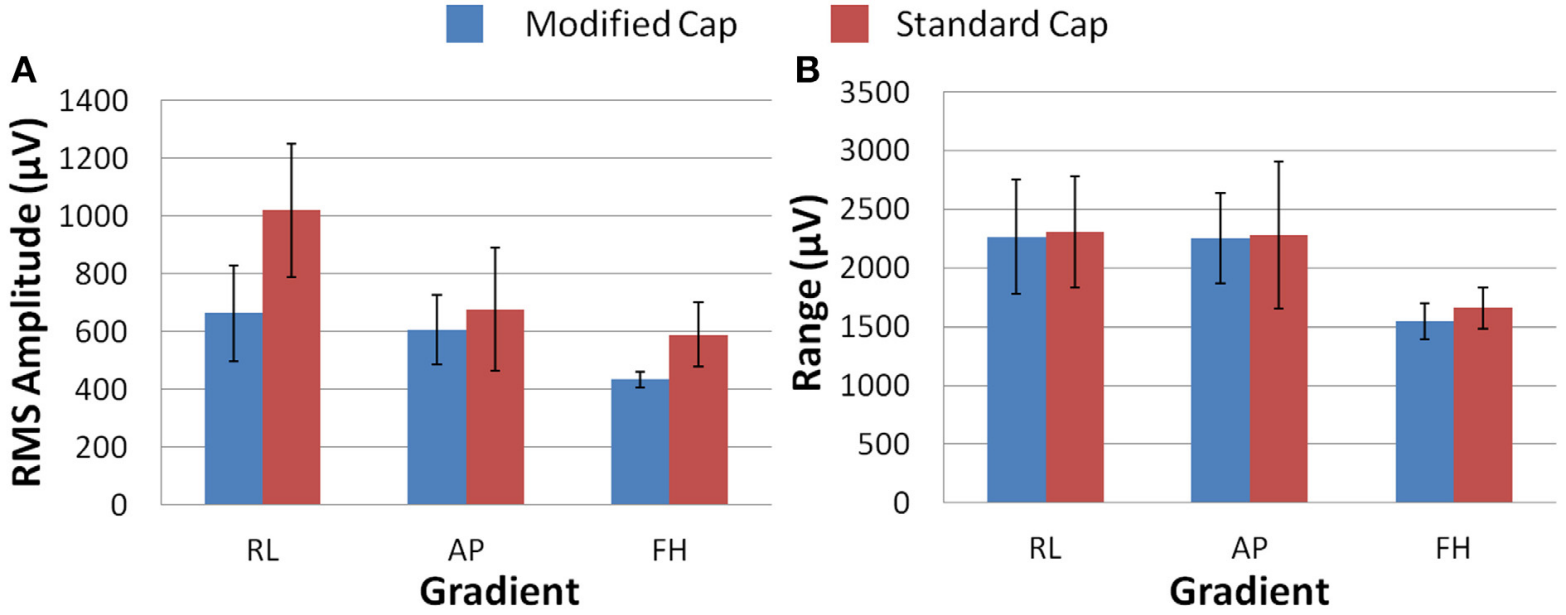
**The average RMS (A) and range (B) of the induced GA over recording repeats for each orthogonal gradient (RL, AP, and FH) on the phantom; for the modified (blue) and standard (red) lead paths**. Error bars show the standard deviation of the measures over repeats.

Figure 6A shows that differences in the induced GA between the cap configurations were observed in the RMS measure on the phantom. The modified cap generated lower induced GA from all three orthogonal gradients compared with the standard cap configuration. However, a considerable variation in this measure between repeated recordings was seen for the RL and AP gradients. This variability suggests that the most consistent gains in performance from use of the modified cap may be for the FH gradient, since the reduction of the GA for this gradient seemed to be less dependent on small changes in position between repeated recordings. Little difference in the range of the GA induced when using the two different cap configurations was observed for any of the three applied gradient directions (Figure 6B).

**Figure 7:**
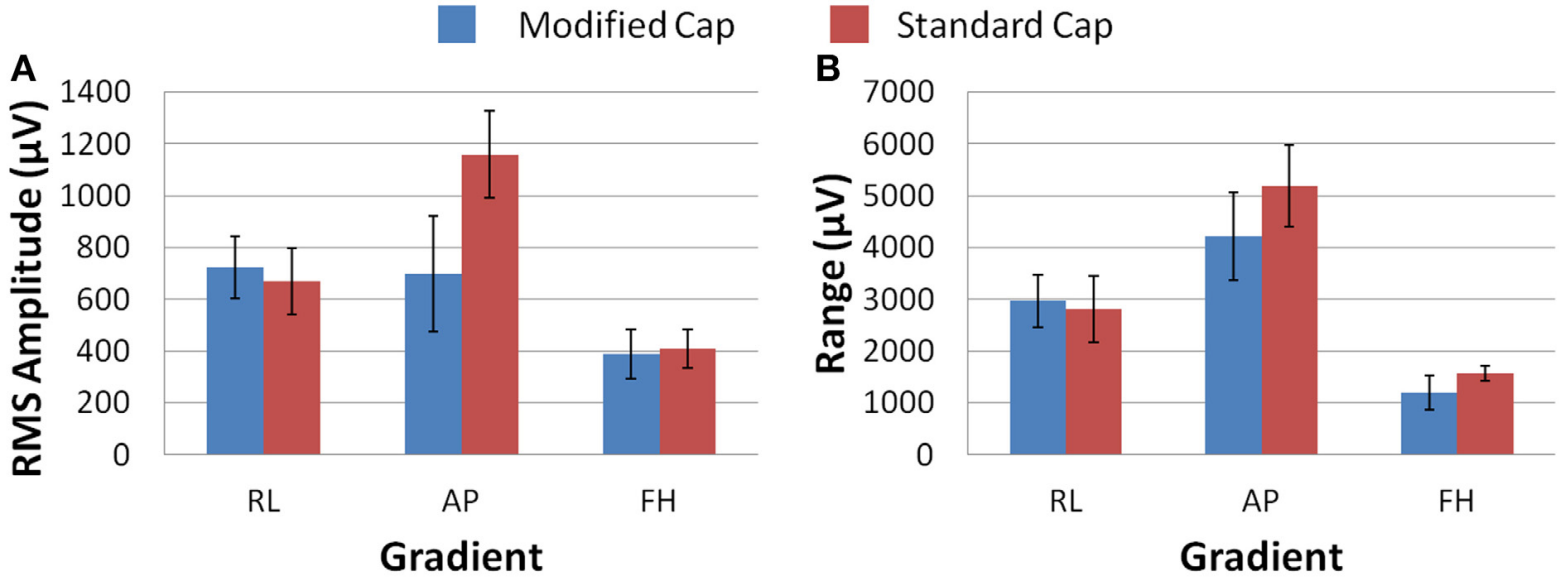
**The average RMS (A) and range (B) of the induced GA over recording repeats and subjects for each orthogonal gradient (RL, AP and FH); for the modified (blue) and standard (red) lead paths**. Error bars show the standard deviation of the measures over subjects.

Figure 7 shows that, on average over all subjects and repeats, the greatest difference between the GA measured with the standard and modified cap on the subject was found to be for the AP gradient. However, as found with the phantom recordings, a high degree of variability was observed over subjects. The reduction in the GA due to the FH gradient was smaller, but more consistent across subjects than that seen for the AP gradient when considering the RMS or range measures. Therefore, the greatest benefit in GA reduction in EEG-fMRI studies of brain function may be for the AP gradient although the gains will vary across subjects. In agreement with the simulations, the RL gradient amplitude (RMS) was found to increase when using the modified cap compared with the standard cap.

#### Study 2: EPI

**Figure 8:**
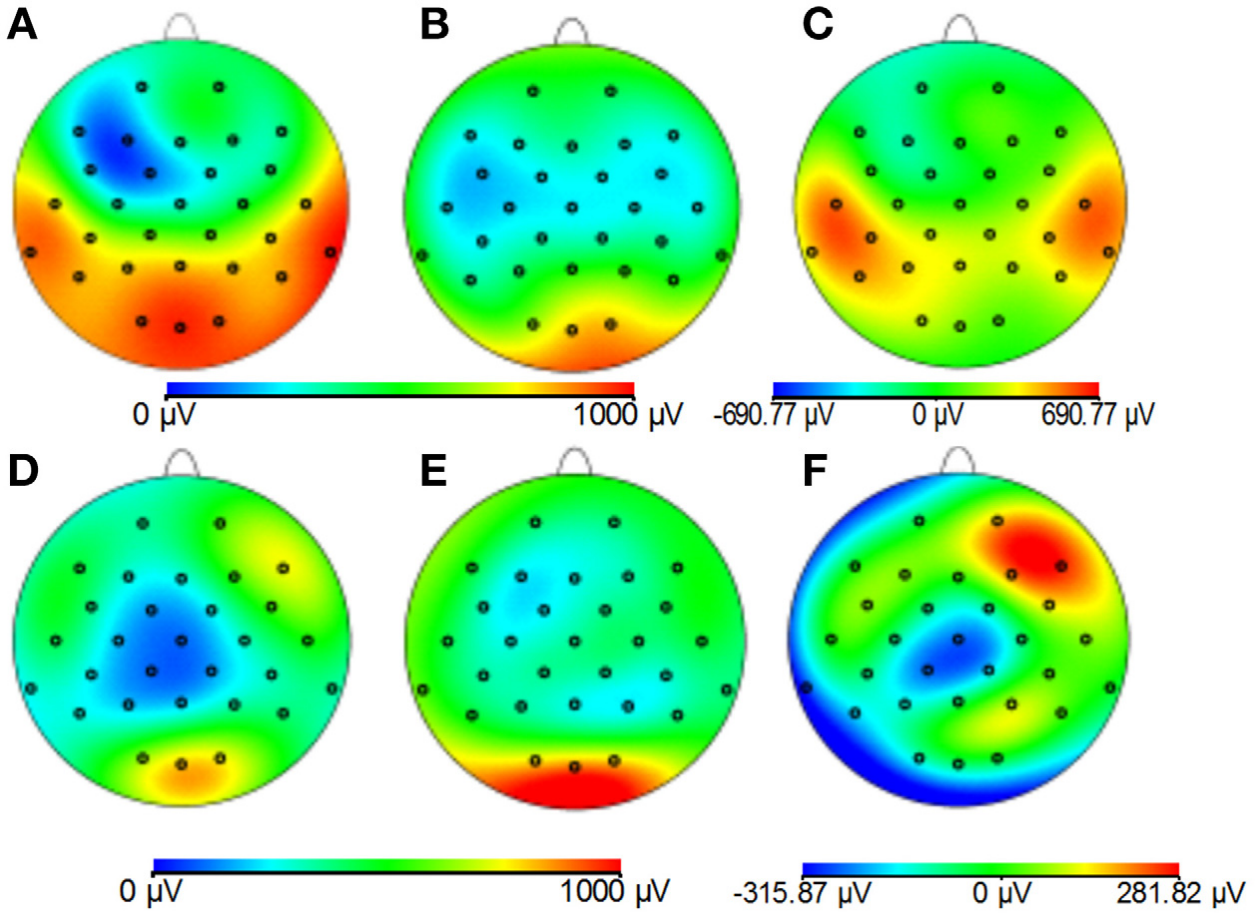
**Maps of the average RMS (over time) of the GA produced by a multi-slice EPI acquisition with the standard cap (A,D); modified cap (B,E) on the phantom (A–C) and the subjects (D–F)**. **(C,F)** Show the difference in the induced GA between caps (**A,B,D,E**, respectively).

Figures 8A–C demonstrates that, for the phantom, the modified cap most significantly reduces the amplitude of the GA due to an EPI sequence over the “temple regions.” This finding is consistent with the results of the simulations (Figure 4). A similar artifact reduction pattern is not seen clearly in the average subject data (Figures 8D–F), although a slight increase in the artifact amplitude is shown in the posterior and anterior regions when the modified cap is used compared with the standard cap, also in agreement with the simulations. The RMS measurement over slice acquisitions on the phantom averaged over all channels and repeats was reduced from 600 ± 100 μV when the standard cap was used to 380 ± 50 μV when the modified cap was used (error denotes the standard deviation across repeats). However, as suggested by Figures 8D–F, this finding was not reflected in the subject data where the average RMS over all repeats, subjects and channels increased from 380 ± 40 μV when the standard cap was employed to 410 ± 60 μV when the modified cap was used (error denotes the standard deviation across subjects). No significant difference in the performance of the caps was found over subjects.

No significant differences were found in the movement parameters identified in the experiments employing the two different caps for either the phantom or subject data. The maximum RMS displacement of the phantom over the data acquisition was 1.5 mm translation (z-direction) and 0.016° rotation (pitch). For the subjects, the maximum displacements were smaller: 0.5 mm translation (z-translation) and 0.005° rotation (pitch). These movements were sufficiently small to allow evaluation of the effect of realistic movements on GA correction for each cap. Using the EEG data collected during the EPI sequence with the movements of the phantom/head enabled interrogation of the effect of the lead and cable configuration on the residual GA after correction using AAS. Using the GA- corrected data, the RMS of the harmonics of the slice repetition frequency averaged over channels and repeats measured on the phantom yielded values of 11 ± 3 μV and 6 ± 2 μV (mean ± standard deviation over repeats) for the standard and modified caps, respectively, suggesting that the variation in the GA is reduced when the modified cap is employed. However, no significant difference between caps was seen for the subject data, which yielded values of 4 ± 2 μV and 5 ± 2 μV (mean ± standard deviation over subjects) for the standard and modified caps, when averaged over subjects, respectively.

## Discussion

Simple consideration of Faraday's Law might suggest that identification of the optimal lead configuration is straightforward since shorter wires would result in smaller loop areas and thus lower induced GAs. However, the interaction of the voltages induced in the volume conductor (the head) with those generated in the leads make this a more complex problem (Yan et al., 2009). Simulations were therefore required to identify the lead configuration that optimally reduces the overall induced GA. These simulations showed that modification of the lead paths and cable bundle position greatly affected the amplitude of the induced GAs. Figure 4, for example shows that a reduction in the GA due to a FH gradient can be achieved for a spherical volume conductor without any change in the cable bundle position by changing from standard lead paths to paths that follow great circles (Figures 4A,B), while Figure 4C shows that a further reduction in this GA can be achieved by moving the cable bundle position along the midline to lie at electrode Cz, rather than between electrodes Cz and Pz. Figure 5A shows that this optimal cap configuration produces a reduction in the range of the GA for all three gradient directions, although the RMS over leads of the artifact is only reduced for the FH gradient (Figure 5B). The simulations also indicated that moving the cable bundle off the midline in the RL direction does not reduce the range or RMS value of the induced GA over leads. This result can probably be explained by the removal of the RL symmetry of the lead arrangements relative to the applied gradients, which occurs when the cable bundle position is moved in the RL direction; this means that although the artifact on some channels is reduced other channels experience a larger increase in the induced GA and as a consequence the range and RMS amplitude of the artifact is not reduced. This strategy might however be employed in studies where the focus is on recording from one side of the head.

The results obtained in the experiments on the spherical phantom in Study 1 largely agree with those found from the simulations, although the modified cap produced a reduction in the range and RMS amplitude of the GAs induced by all three gradients (Figure 6). However, the results of similar experiments on human subjects were significantly different, with the modified cap producing little reduction in the artifact induced by a FH gradient with a much larger reduction for the AP gradient, and an increase in the range and RMS amplitude of the GA due to a RL gradient (Figure 7).

The results of the simulations and the experimental measurements of Study 1 thus clearly show that the effect of the lead configuration on the induced GA during a standard EPI sequence will be strongly dependent on the image geometry, which dictates the direction of the gradients used in the gradient waveform elements that cause the dominant temporal features of the GA. It is known that when a 250 Hz low-pass cut-off is employed, the periods of gradient switching of a standard EPI sequence which produce the largest elements of the GA are the slice select, phase-encode pre-excursion and crusher gradient pulses (Mullinger et al., 2008b). The orientation of the slices and the phase-encode direction determine the directions in which the slice select and phase-encode pre-excursion gradient pulses are applied. fMRI data acquisitions generally employ axial slice geometry and the phase-encoding direction is generally AP, so that the image distortions due to field inhomogeneity do not disturb the left-right symmetry of the brain. With this image geometry, which was used in Study 2, the slice selection gradients are applied in the FH direction, while the phase-encode pre-excursion pulse is applied in the AP direction. The crusher gradient pulses generally employ all three orthogonal gradient channels so as to maximize signal dephasing.

Since Study 1 showed that the reductions in the RMS of the GA induced on the spherical phantom could be achieved with the modified cap for the AP and FH gradients (Figure 6), we would predict that the GA induced by the EPI sequence employed in Study 2 would also be reduced by the modified cap design. This was found to be the case with the RMS of the GA observed on the phantom being reduced from 600 ± 100 μV to 380 ± 50 μV by use of the modified cap. Unfortunately, a similar reduction was not reflected in the subject data where in fact no significant change in the induced GA was measured across subjects and repeated measures when comparing data from the standard and modified caps. Maps of the average RMS (over time) of the GA on the phantom due to the EPI sequence (Figures 8A–C) show that the modified lead configuration reduced the GA amplitude to the greatest extent in the “temple regions.” Since the simulations showed that the modified lead paths result in the largest reduction in the GA over temple areas when a FH gradient was used (Figure 4) it is likely that the pattern of GA reduction measured on the phantom is primarily accounted for by the FH gradient, employed during slice selection. The lack of a reduction in the RMS of the GA induced by the FH gradient on the human subjects (Figure 7) may therefore explain why no significant reduction of the GA was seen when the EPI sequence was applied to human subjects. Given the lack of a significant change in the induced GA on the subjects it is unsurprising that GA correction using AAS did not perform significantly better when the modified cap was used rather than the standard one.

The observed difference in the performance of the modified cap in reducing the GA for the spherical phantom and human subjects most likely results from differences in volume conductor geometry. Whilst the spherical phantom used in the experimental work matched the geometry used in the simulations, this was obviously not the case for the subjects' heads, which varied in shape across subjects and always deviated from sphericity. Consequently the lead paths did not form great circles when the EEG cap was placed on the subjects' heads and it is likely that the leads serving electrodes over the temple regions were most distorted from great circle paths, because of the flat sides of the head. This distortion may explain why the induced GA was not reduced for data acquired on the human subjects in the same way as on the phantom. To test this hypothesis, the recordings described in Study 1 were repeated for the two cap arrangements using a head-shaped agar phantom (see Figure 2) formed using a special mold. Figure S1 shows the results. A strong similarity can be seen between the results obtained on the head-shaped phantom with those recorded from the subjects' heads (Figure 7 vs. Figure S1). The GA induced by the FH gradient shows no clear difference between the two caps whilst the GA from the AP gradient is considerably smaller for the modified cap than for the standard cap. This provides a strong indication that the discrepancy in the data acquired on the subjects compared with that from the spherical phantom probably results from differences in the shape of the volume conductors onto which the caps are placed.

The lesser differences between the results of the simulations and the experiments on the spherical phantom are likely to be caused by small errors in lead path positioning resulting from the difficulties of cap construction. In addition, there will be some contribution to the GA from the wires in the cable bundle and those in the cable between the EEG cap connector and the amplifier, as previously discussed (Yan et al., 2009). Whilst for this work we removed the ribbon cable and replaced it with a cable bundle so as to minimize the GA induced in this cabling, as described by Yan et al. (2009), it is not possible using a conventional EEG setup to remove all contributions of the cabling to the observed GA. The relatively large standard deviations of the RMS and range measures shows how sensitive the induced GA is to external factors such as positioning of cables and leads (Mullinger et al., 2011). The variance in the measures for the phantom data (Figure 6) provides an indication of the sensitivity of the induced GA to the exact lead and cable positions. The standard deviation of the RMS over repeated measures is reduced for all three gradients when using the modified cap compared with the standard one. This reduction in variability of the GA is translated into an improvement in GA removal when using AAS with the modified cap on the phantom compared with the standard cap. The standard deviation over subjects is not only sensitive to variations in subject positioning in the scanner but also to the different head shapes, which produce different wire paths. Therefore, the increase in standard deviation when the modified cap is used is most likely be due to a mixture of these effects and cannot easily be directly linked to the performance of AAS for GA correction. The smaller residual GAs in the subject data compared with the phantom are most likely to be due to the movements of the subjects' heads being less than those applied to the phantom in Study 2 (Moosmann et al., 2009).

The large differences observed between the induced GA in the simulations and experimental results in Study 1 when using a head (or head-shaped phantom) compared with the relative similarity when using a spherical phantom, suggest that wire path optimization is highly dependent on the volume conductor geometry. Therefore, further simulations are required to ascertain the optimal lead configuration for a volume conductor with more realistic head-shaped geometry. As in this study, the electrode positions on the head could again be defined by digitizing the electrodes on a head. However, these electrode positions would no longer be projected onto a sphere, thus violating the assumptions made to calculate the scalar potential employing an analytical solution (Yan et al., 2009) which was used here. Consequently numerical analysis would be needed to calculate the contribution of the scalar potential term of the GA. This may be achieved using methods recently employed by Chowdhury et al. (2013). Wire paths would follow the contours of the head rather than great circles, with a similar method of varying the position of the cable bundle to that used in this study. These measures should provide a more accurate estimate of the overall GA induced on the head by different wire configurations from which to evaluate the potential gain of changing the wire paths on the EEG cap for EPI sequences with different image geometries (axial, coronal or sagittal slices) where different gradients (RL, AP, and FH) dominate the induced GA. We hypothesize that these advanced simulations would show that for the optimal wire configuration tested in this work (cable bundle position at Cz), the greatest reduction in GA amplitude would occur for the AP gradient (as shown in the experimental data on subjects, Figure 7) with the variation of the artifacts with cable bundle position differing from that shown in Figure 3.

In future work, simulations using realistic head-shaped models could also be used to investigate wire configurations which optimally minimize sensitivity to changes in GA due to changes in head position. Previous work has shown that the GA varies linearly with small changes in head position (Yan et al., 2009). If possible, minimizing the rate of change of the GA with position, as well as the overall amplitude of the GA targeted in this work, would further improve the quality of EEG data recorded during concurrent fMRI. However, this issue is non-trivial as there will be interactions between the angulations of the head, the type of movement and head-shape; therefore further investigation is required to identify if it is possible to find a single cable configuration which reduces the change in GA for all types of head movement and all directions of gradient (RL, AP and FH) taking account of sensitivity to head geometry, which will vary on a subject by subject basis.

The work described here shows that it is possible to reduce the induced GA by changing the lead and cable bundle positions. However, the experimental work indicates that use of a simple spherical model of the head in identifying the optimal position of the cable bundle yields a cap design which does not reduce the GA significantly when used on human subjects. Further work is needed to assess whether the use of a more realistically head-shaped volume conductor in the optimization process will yield a cap design that reduces the GA on average in human subjects. With the set-up tested here, the greatest reductions in the GA on the spherical phantom were seen over the “temple regions” which would be particularly useful in studies focusing on auditory responses. Our results also suggest that other lead configurations could be used to minimize the GA at different electrodes depending on the cortical areas of interest. Although the subject data did not reflect the reductions over the “temple regions,” small modifications of the lead paths may allow such a reduction to be achieved. The resulting cap might then also be beneficial in reducing the pulse artifact which is largest over the temple regions (Debener et al., 2008; Yan et al., 2010).

## Author contributions

Simulations and experiments were designed by Karen J. Mullinger and Richard Bowtell. Simulations were performed by Karen J. Mullinger. Experimental data were acquired by Karen J. Mullinger and Muhammad E. H. Chowdhury and analyzed by Karen J. Mullinger. All authors were involved in interpretation of data and paper writing.

### Conflict of interest statement

The authors declare that the research was conducted in the absence of any commercial or financial relationships that could be construed as a potential conflict of interest.
