# Supplementary material for: Investigating the effect of modifying the EEG cap lead configuration on the gradient artifact in simultaneous EEG-fMRI
Source: Front Neurosci. 2014 Jul 29;8:226. doi: 10.3389/fnins.2014.00226 (PMC4114285; doi:10.3389/fnins.2014.00226)
Supplement: Supplementary file 1 [file DataSheet1.DOCX]

***Supplementary Material***

**Investigating the effect of modifying the EEG cap lead configuration on the gradient artefact in simultaneous EEG-fMRI**

**Karen J. Mullinger^1,2*^, Muhammad E.H. Chowdhury^1^ and Richard Bowtell^1^**

^1^Sir Peter Mansfield Magnetic Resonance Centre, School of Physics and Astronomy, University of Nottingham, University Park, Nottingham, NG7 2RD, UK.

^2^Birmingham University Imaging Centre, School of Psychology, University of Birmingham, Birmingham, UK

*** Correspondence:** Dr Karen Mullinger, Sir Peter Mansfield Magnetic Resonance Centre, School of Physics and Astronomy, University of Nottingham, Nottingham, NG7 2RD, UK.

Email: karen.mullinger@nottingham.ac.uk

**Supplementary Figure**

*Supplementary Figure S1:* The average RMS (A) and range (B) of the induced GA over recording repeats for each orthogonal gradient (RL, AP and FH) on the head shaped phantom; for the modified (blue) and standard (red) lead paths. Error bars show the standard deviation of the measures over repeats.
